# Gestational high‐fat diet impaired demethylation of *Ppar* *α* and induced obesity of offspring

**DOI:** 10.1111/jcmm.16551

**Published:** 2021-05-06

**Authors:** Haiyan Pang, Dandan Ling, Yi Cheng, Rubab Akbar, Luyang Jin, Jun Ren, Haiyan Wu, Bin Chen, Yin Zhou, Hong Zhu, Yuzhong Zhou, Hefeng Huang, Jianzhong Sheng

**Affiliations:** ^1^ Department of Reproductive Medicine Women’s Hospital School of Medicine Zhejiang University Hangzhou China; ^2^ The Key Laboratory of Reproductive Genetics （Zhejiang University School of Medicine), Ministry of Education Hangzhou China; ^3^ Hospital of Obstetrics and Gynecology Fudan University Shanghai Medical College Shanghai China; ^4^ Department of Pathology and Pathophysiology School of Medicine Zhejiang University Hangzhou China; ^5^ Assisted Reproduction Unit Department of Obstetrics and Gynecology Sir Run Run Shaw Hospital School of Medicine Zhejiang University Hangzhou China; ^6^ Center for Reproductive Medicine School of Medicine the Second Affiliated Hospital Zhejiang University Hangzhou China

**Keywords:** DNA demethylation, high‐fat diet, lipid metabolism, normal chow diet, obesity

## Abstract

Gestational and postpartum high‐fat diets (HFDs) have been implicated as causes of obesity in offspring in later life. The present study aimed to investigate the effects of gestational and/or postpartum HFD on obesity in offspring. We established a mouse model of HFD exposure that included gestation, lactation and post‐weaning periods. We found that gestation was the most sensitive period, as the administration of a HFD impaired lipid metabolism, especially fatty acid oxidation in both foetal and adult mice, and caused obesity in offspring. Mechanistically, the DNA hypermethylation level of the nuclear receptor, peroxisome proliferator‐activated receptor‐α (*Pparα*), and the decreased mRNA levels of ten‐eleven translocation 1 (*Tet1*) and/or ten‐eleven translocation 2 (*Tet2*) were detected in the livers of foetal and adult offspring from mothers given a HFD during gestation, which was also associated with low *Pparα* expression in hepatic cells. We speculated that the hypermethylation of *Pparα* resulted from the decreased *Tet1/2* expression in mothers given a HFD during gestation, thereby causing lipid metabolism disorders and obesity. In conclusion, this study demonstrates that a HFD during gestation exerts long‐term effects on the health of offspring via the DNA demethylation of *Pparα*, thereby highlighting the importance of the gestational period in regulating epigenetic mechanisms involved in metabolism.

## 1 INTRODUCTION

Obesity is a major health issue worldwide, with excessive fat intake contributing to disease progression. Increasing evidence shows that obesity increases the risk of developing type 2 diabetes, non‐alcoholic fatty liver disease and cardiovascular disease, thereby reducing the quality of life and life expectancy of patients.^1^, ^2^ However, it remains unknown whether there is a critical period during development from the gamete to the adult, and whether there is a relationship between high‐fat diet (HFD)‐induced obesity and gender. Nonetheless, maternal pre‐pregnancy and gestational obesity have adverse health outcomes in offspring, and this positive relationship has been confirmed in several studies.^3^, ^4^ The effects of a HFD given to mothers during lactation on metabolism in offspring are still controversial.^5^, ^6^ Presently, a wealth of food choices has resulted in bad eating habits, suggesting that changing environments may lead to adverse health outcomes in adults, which is a major driver of the obesity epidemic. However, it is unknown which period is most critical on the metabolism of offspring.

The pathogenesis of obesity not only includes various genetic factors, but also social and obesogenic environmental factors.^5^, ^7^ One study has investigated the epigenetic signature related to obesity in later life.^8^ However, there is little information on the specific mechanisms of metabolic programming, including when and how epigenetic changes occur. DNA methylation is an important epigenetic hallmark, and DNA methylation patterns of genes are often altered by deleterious environmental factors. Furthermore, DNA methylation levels are regulated by DNA‐modifying enzymes. The ten‐eleven translocation (TET) family is comprised of important DNA‐modifying enzymes with roles in the epigenetic regulation of genes.^9^ TETs promote DNA demethylation and increase gene expression. DNA demethylation occurs in foetal and adult livers and associates with the fatty acid β‐oxidation genes.^10^ However, the major challenge is whether we can gauge the critical timing and the effects of HFD that can contribute to obesity.

In the present study, we established a mouse model to identify the most sensitive stage of obesity development in response to HFD. Furthermore, we confirmed its possible mechanism of action in obese offspring.

## 2 MATERIALS AND METHODS

### 2.1 Animal care

All animal protocols were approved by the Zhejiang University Animal Care and Use Committee. All mice were purchased from Beijing Weitonglihua Laboratory Animal (Beijing, China). The mouse HFD model was established as shown in Figure S1. Virgin C57BL/6J females (age, 6 weeks; weight, 12 g) were purchased and provided either a HFD (#D12492’ Research Diets, containing 20% protein, 60% fat) or a normal chow diet (NCD) (#D12450B; Research Diets, containing 20% protein, 10% fat) for 8 weeks before mating. Mice were provided either a NCD or a HFD during pre‐pregnancy and pregnancy. C57BL/6J male mice (age, 10 weeks) were used for mating. After birth, each group was divided into two groups: NCD and HFD groups based on the food provided to the mothers. After weaning, offspring in each group (four groups in total) was further divided into two groups: NCD and HFD groups based on the food provided to the offspring. This resulted in eight groups of offspring of two sexes, and there is only one pup from the same litter in each group (Figure S1).

### 2.2 Serum biochemical measurements

Blood specimens were collected from 16‐week‐old mice after overnight fasting. The serum levels of fasting triglyceride (TG), total cholesterol (TC), high‐density lipoprotein (HDL), low‐density lipoprotein (LDL) and non‐esterified fatty acids (NEFA) were measured using a biochemical analyser (TBA120FR; Toshiba, Tokyo, Japan). The serum level of fasting insulin was quantified using a mouse insulin enzyme‐linked immunosorbent assay (Crystal Chem, Downers Grove, IL, USA). The fasting glucose level was measured using a biochemical analyser (TBA120FR; Toshiba). After euthanization, livers and free gonadal adipose tissues were harvested and measured to calculate the liver‐to‐weight ratio and gonadal fat‐to‐weight ratio.

### 2.3 Tissue collection, histology and analysis of gene expression

Livers were harvested after collecting blood. For lipid accumulation analysis, left liver lobes were fixed, embedded in paraffin and sectioned for staining with Oil Red O. Total RNA from livers was extracted using TRIzol reagent (Life Technologies, Grand Island, NY, USA). cDNA was synthesized using oligo‐dT and random primers (Takara, Tokyo, Japan). The levels of target genes were measured using quantitative real‐time polymerase chain reaction (PCR) (LightCycler 480 II; Roche, Switzerland) with SYBR green detection (Takara). The level of each target gene was normalized to the β‐actin level. All primer (Sangon Biotech, Shanghai, China) sequences are shown in Table S1.

### 2.4 Pyrosequencing

DNA was extracted from livers using the TIANmap DNA kit (Tiangen, Beijing, China) and converted to bisulphite using the EpiTect Bisulfite Kit (Qiagen, Dusseldorf, Germany) according to the manufacturer's instructions. Pyrosequencing was used to assess the methylation status of gene promoter regions. Methylation pyrosequencing primers were designed by PyroMark Assay Design software 2.0 (Qiagen). Agarose gel electrophoresis was used to analyse each PCR product. PCR was performed in a 12.5 μL final volume that included EpiTaq Hot Start DNA polymerase (Takara, Dalian, China). For pyrosequencing analysis, bisulphite PCR products were processed according to the manufacturer's instructions (Qiagen). In brief, 10 μL of the PCR product was immobilized on 5 μL of streptavidin Sepharose high‐performance beads (GE Healthcare, Chicago, IL, USA) and annealed to 1 μL of a sequencing primer (final concentration, 10 μM) for 2 minute at 80°C. Pyrosequencing was performed using a PSQ 96MA instrument (Qiagen) using appropriate enzymes, substrates and nucleotides (PyroMark Gold Q24 reagents; Qiagen). CpG analysis was performed using PSQ 96MA software. Primer sequences and positions of validated CpG sites are shown in Table S2.

### 2.5 Statistical analysis

Statistical analysis was performed using SPSS Statistics 20.0 software (IBM, Armonk, NY, USA) and GraphPad Prism 6.0 software (La Jolla, CA, USA). All data are presented as the mean ± standard error of the mean. Unpaired Student's *t* tests were used to analyse statistical significance between two independent groups. One‐way analysis of variance followed by the least significance difference post hoc test was used to analyse statistical significance among three or more groups. All reported *P* values are two‐sided. *P* < .05 was considered statistically significant.

## 3 RESULTS

### 3.1 Administration of a HFD during gestation affects lipid metabolism and causes obesity in offspring

**FIGURE 1:**
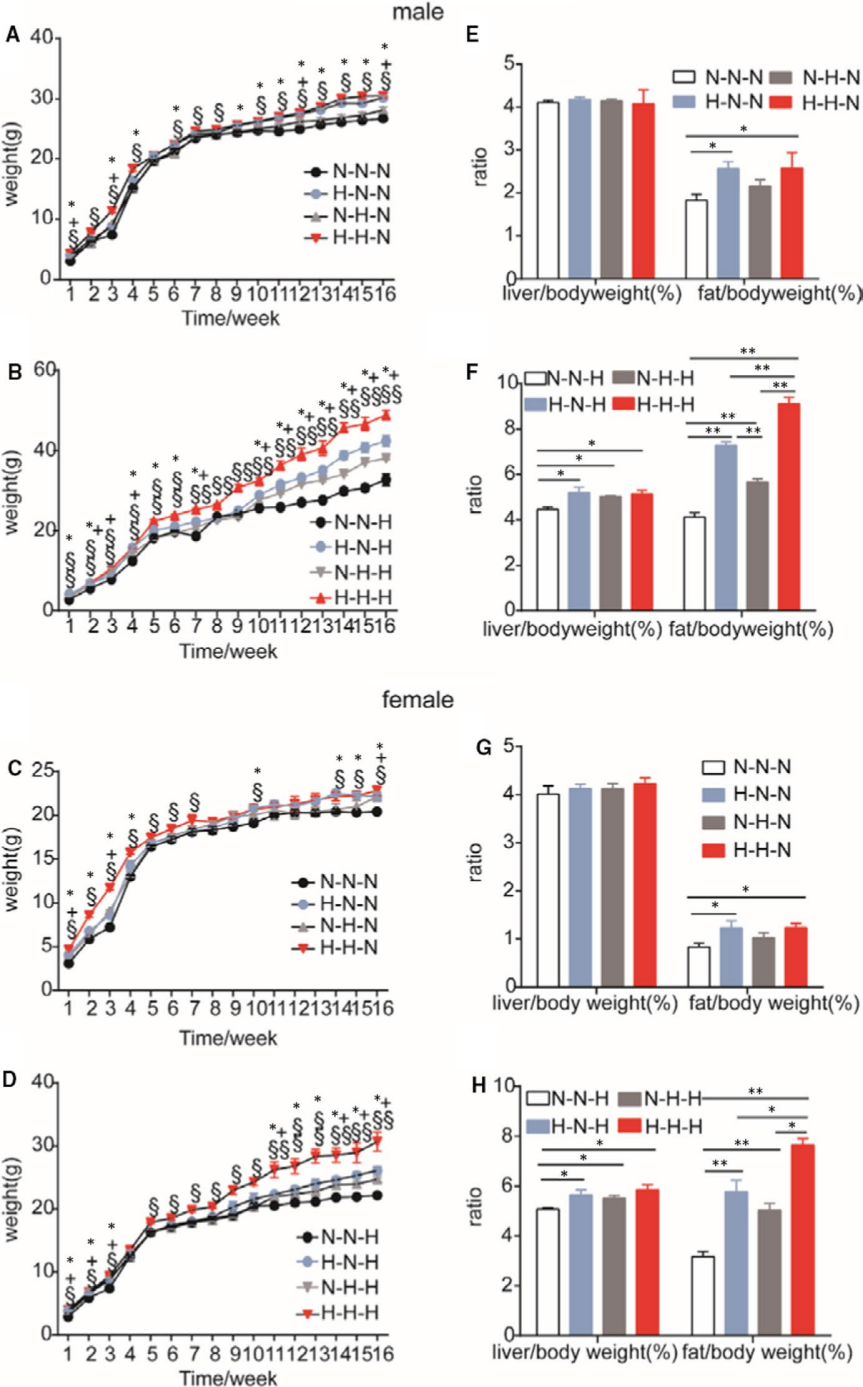
Offspring growth curves, liver‐to‐weight ratios and gonadal fat‐to‐weight ratios in F1 offspring at 16 weeks of age. A, bodyweight of F1 male offspring on a normal chow diet or B, high‐fat diet after weaning; C, bodyweight of F1 female offspring on a normal chow diet or D, high‐fat diet after weaning. A, N–N–N = 10 vs H–N–N = 8 vs N–H–N = 11 vs H–H–N = 9; B, N–N–H = 10 vs H–N–H = 9 vs N–H–H = 8 vs H–H–H = 12; C, N–N–N = 8 vs H–N–N = 8 vs N–H–N = 8 vs H–H–N = 9; D, N–N–H = 13 vs H–N–H = 10 vs N–H–H = 9 vs H–H–H = 10. Liver‐to‐weight ratios and gonadal fat‐to‐weight ratios in F1 male offspring on E, a normal chow diet (N–N–N = 10 vs H–N–N = 8 vs N–H–N = 11 vs H–H–N = 9) or F, high‐fat diet (N–N–H = 10 vs H–N–H = 9 vs N–H–H = 8 vs H–H–H = 12) after weaning; liver‐to‐weight ratios and gonadal fat‐to‐weight ratios in F1 female offspring on G, a normal chow diet (N–N–N = 8 vs H–N–N = 8 vs N–H–N = 8 vs H–H–N = 9) or H, high‐fat diet (N–N–H = 13 vs H–N–H = 10 vs N–H–H = 9 vs H–H–H = 10) after weaning. Data are presented as the mean ± SEM *N–N–N vs H–N–N, ^+^N–N–N vs N–H–N, ^§^N–N–N vs H–H–N, *N–N–H vs H–N–H, ^+^N–N–H vs N–H–H, ^§^N–N–H vs H–H–H. *P < .05, **P < .01, significance was determined by ANOVA

We analysed the offspring that were given a HFD during gestation, during weaning and after weaning. Irrespective of whether the offspring were given a NCD or HFD after weaning, the offspring that were given a HFD during gestation had a significantly increased bodyweight compared to the offspring of the other groups at 16 weeks of age, and there were no sex‐specific differences in chance of obesity (Figure 1A–D, one‐way analysis of variance [ANOVA], *P* < .05). Among the four groups given a HFD after weaning, the weight gain was most obvious in the groups given a HFD during gestation (H‐H–H and H–N–H, Figure 1B, D, one‐way ANOVA, *P* < .05).

### 3.2 Administration of a HFD increased liver lipid accumulation and ectopic fat deposition in offspring

**FIGURE 2:**
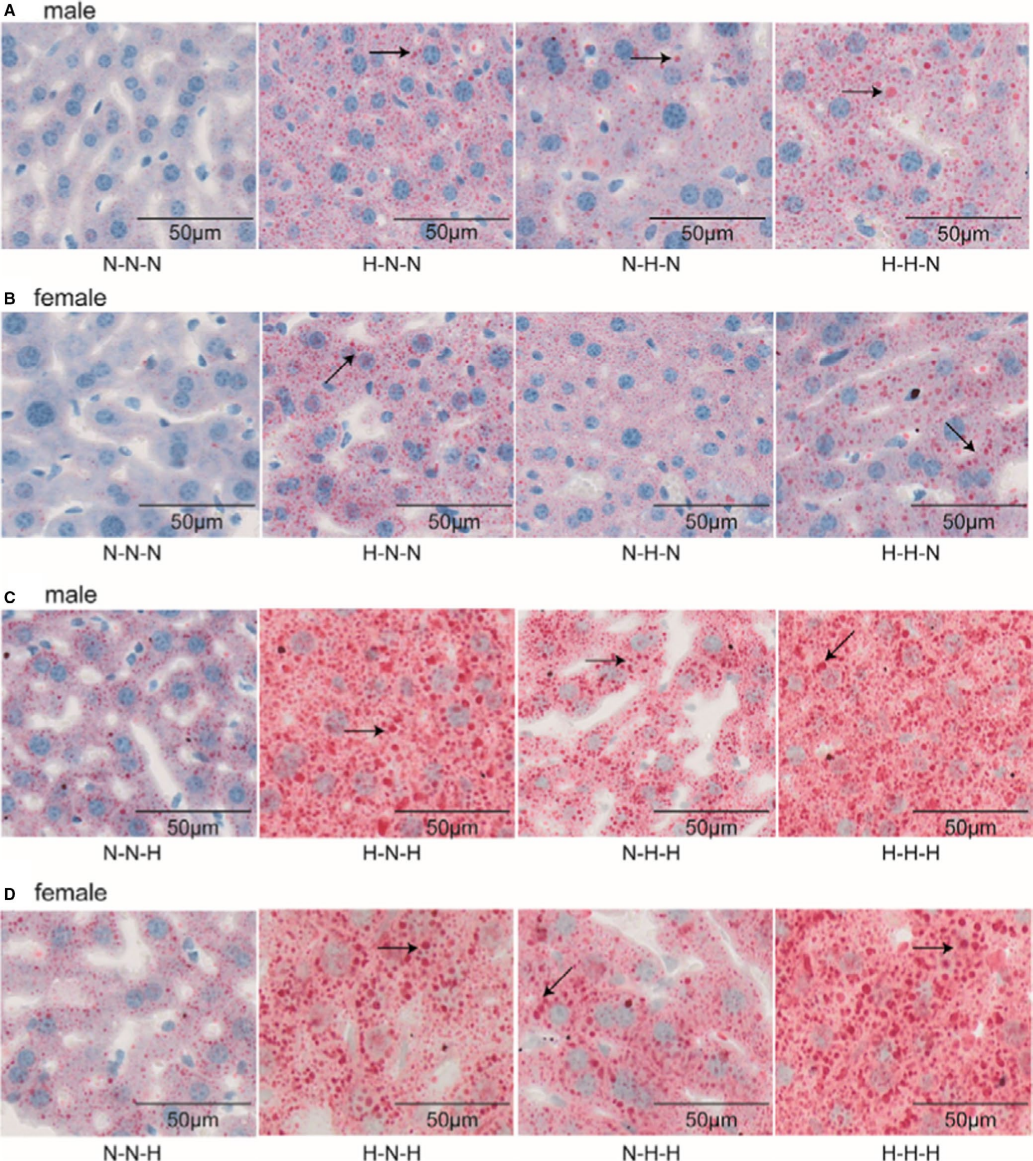
Oil Red O‐stained liver cross‐sections showing fat accumulation in F1 offspring mice at 16 weeks of age. A, administration of a NCD after weaning and C, a HFD after weaning in male mice; B, administration of a NCD after weaning and D, a HFD after weaning in female mice

A hallmark feature of obesity is ectopic fat deposition. Lipid accumulation was observed in livers of mice of all groups, except the N–N–N group (Figure 2 and Supplemental Figure S2). More and bigger lipid droplets were observed in livers of offspring given a HFD after weaning (Figure 2C [male], D [female]) compared with livers of offspring given a NCD after weaning (Figure 2A [male], B [female])) at 16 weeks of age. An analysis of the liver‐to‐bodyweight ratio showed that there was no significant difference in male and female offspring given a NCD feeding after weaning (Figure 1E, G, one‐way ANOVA, *P* > .05). However, offspring given a HFD after weaning (groups H–N–H, N–H–H and H–H–H) showed significantly increased liver‐to‐bodyweight ratios compared with offspring given a NCD (group N–N–H), irrespective of whether the offspring were male (Figure 1F) or female (Figure 1H, one‐way ANOVA, *P* < .05). Furthermore, we evaluated the gonadal fat‐to‐bodyweight ratio. An analysis of the gonadal fat‐to‐weight ratio showed that in four NCD after‐weaning groups, all male and female offspring in H–N–N and H–H–N groups had significantly increased gonadal fat‐to‐weight ratio compared with the control group (N–N–N) (Figure 1E, G). In four HFD after‐weaning groups, all male and female offspring in H–N–H, N–H–H and H–H–H groups had significantly increased gonadal fat‐to‐weight ratios compared with the control group (N–N–H) (Figure 1F, H, one‐way ANOVA, *P* < .05). However, offspring of gestational HFD exposure groups, including H–N–H and H–H–H groups, had significantly higher gonadal fat‐to‐weight ratios than offspring in the N–H–H group (Figure 1F, H). Taken together, these results indicate that a HFD given during gestation contributes to bodyweight gain and ectopic fat deposition in offspring mice.

### 3.3 Administration of HFD altered serum TG, LDL, NEFA, fasting glucose and insulin levels in offspring

Given that the important hallmarks of obesity are metabolic disorders and insulin resistance, we measured serum TG, TC, LDL, HDL, NEFA, glucose and insulin levels in offspring. Compared with the control group (N–N–N), offspring exposed to a HFD during gestation or lactation showed increased TG, LDL, NEFA, fasting glucose and insulin levels (Table 1). Offspring exposed to a HFD after weaning (H–N–H, N–H–H and H–H–H groups) showed very significantly increased TG, LDL, NEFA, fasting glucose and insulin levels and a decreased HDL level (Table 2). These results indicate that mice exposed to a HFD during early life can develop lipid and glucose metabolism disorders that may increase the risk of obesity in later life.

**TABLE 1:** Metabolic parameters in offspring mice at 16 weeks of age

	N–N–N	H–N–N	N–H–N	H–H–N
Male (n)	10	8	11	9
TG (mmol/L)	0.87 ± 0.07	1.34 ± 0.09*	1.34 ± 0.11*	1.44 ± 0.09*
TC (mmol/L)	2.02 ± 0.05	2.11 ± 0.04	2.05 ± 0.05	2.08 ± 0.05
LDL (mmol/L)	0.21 ± 0.04	0.4 ± 0.07*	0.31 ± 0.06*	0.38 ± 0.08*
HDL (mmol/L)	1.7 ± 0.34	1.66 ± 0.04	1.63 ± 0.03	1.64 ± 0.06
NEFA (mmol/L)	0.78 ± 0.06	1.09 ± 0.04*	0.98 ± 0.07*	1.08 ± 0.05*
Glucose (mmol/L)	3.89 ± 0.13	5.77 ± 0.24*	6.7 ± 0.51*	6.32 ± 0.28*
Insulin (μIU/mL)	11.55 ± 1.03	17.89 ± 1.02*	14.04 ± 1.64^#^	18.03 ± 0.81^*^, ^$^
Female (n)	8	8	8	9
TG (mmol/L)	0.66 ± 0.05	0.96 ± 0.08*	0.9 ± 0.03*	1.04 ± 0.07*
TC (mmol/L)	1.96 ± 0.02	1.92 ± 0.05	1.94 ± 0.08	2.07 ± 0.04^#^
LDL (mmol/L)	0.15 ± 0.01	0.21 ± 0.01*	0.20 ± 0.01*	0.26 ± 0.02^*^, ^#^, ^$^
HDL (mmol/L)	1.46 ± 0.03	1.23 ± 0.05*	1.28 ± 0.07*	1.16 ± 0.02*
NEFA (mmol/L)	0.67 ± 0.05	1.01 ± 0.05*	0.79 ± 0.12*	1.19 ± 0.08*
Glucose (mmol/L)	4.87 ± 0.24	6.49 ± 0.24*	6.38 ± 0.21*	6.48 ± 0.17*
Insulin (μIU/mL)	14.71 ± 1.14	23.58 ± 1.32*	23.98 ± 3.17*	23.94 ± 2.07*

Note

All parameters were measured at 16 weeks of age. All results are expressed as the mean ± SEM Significance was determined by one‐way ANOVA, followed by LSD post hoc test.

Abbreviations: HDL, high‐density lipoprotein; LDL, low‐density lipoprotein; NEFA, non‐esterified fatty acids; TC, total cholesterol; TG, triacylglycerol.

* *P < *.05.

^#^ *P < *.05.

^$^ *P < *.05.

**TABLE 2:** Metabolic parameters in offspring mice at 16 weeks of age

	N–N–H	H–N–H	N–H–H	H–H–H
Male (n)	10	9	8	12
TG (mmol/L)	1.35 ± 0.10	1.83 ± 0.12*	1.74 ± 0.10*	2.00 ± 0.09*
TC (mmol/L)	2.85 ± 0.11	3.73 ± 0.23*	3.67 ± 0.21*, ^#^	4.84 ± 0.13*, ^$^
LDL (mmol/L)	0.19 ± 0.02	0.39 ± 0.03*	0.40 ± 0.02*	0.56 ± 0.05*, ^#^, ^$^
HDL (mmol/L)	2.56 ± 0.20	1.86 ± 0.23*	2.00 ± 0.20*	1.69 ± 0.07*
NEFA (mmol/L)	1.42 ± 0.06	1.86 ± 0.08**	1.85 ± 0.09**	2.24 ± 0.10**, ^##^, ^$$^
Glucose (mmol/L)	7.28 ± 0.36	11.12 ± 0.51**	10.64 ± 0.47**, ^#^	13.34 ± 0.66**, ^##^, ^$$^
Insulin (μIU/mL)	19.09 ± 0.44	30.68 ± 1.43**	29.96 ± 2.67**, ^#^	32.83 ± 3.49**, ^$^
Female (n)	13	10	9	10
TG (mmol/L)	1.16 ± 0.07	1.46 ± 0.10*	1.46 ± 0.11*, ^#^	1.57 ± 0.09*, ^$^
TC (mmol/L)	2.42 ± 0.08	2.67 ± 0.14	2.52 ± 0.14	3.16 ± 0.11**, ^##^, ^$$^
LDL (mmol/L)	0.78 ± 0.02	0.92 ± 0.04*	0.92 ± 0.03*	1.02 ± 0.08*, ^#^, ^$^
HDL (mmol/L)	1.82 ± 0.04	1.58 ± 0.08*	1.59 ± 0.09*	1.34 ± 0.06*, ^#^, ^$^
NEFA (mmol/L)	1.23 ± 0.04	1.68 ± 0.06**	1.53 ± 0.08**	1.75 ± 0.04**, ^$$^
Glucose (mmol/L)	6.70 ± 0.17	8.04 ± 0.23*	8.00 ± 0.36*	10.25 ± 0.62**, ^##^, ^$$^
Insulin (μIU/mL)	23.94 ± 2.07	30.73 ± 0.93**	29.17 ± 1.04*	30.64 ± 1.27**

Note

All parameters were measured at 16 weeks of age. All results are expressed as the mean ± SEM Significance was determined by one‐way ANOVA, followed by LSD post hoc test.

Abbreviations: HDL, high‐density lipoprotein; LDL, low‐density lipoprotein; NEFA, non‐esterified fatty acids; TC, total cholesterol; TG, triacylglycerol.

* *P* < .05

** *P* < .01 vs N–N–H;

^#^ *P* < .05

^##^ *P* < .01 vs H–N–H ;

^$^ *P* < .05

^$$^ *P* < .01 vs N–H–H.

### 3.4 Administration of a HFD down‐regulated *Pparα* mRNA expression in offspring at 16 weeks of age

**FIGURE 3:**
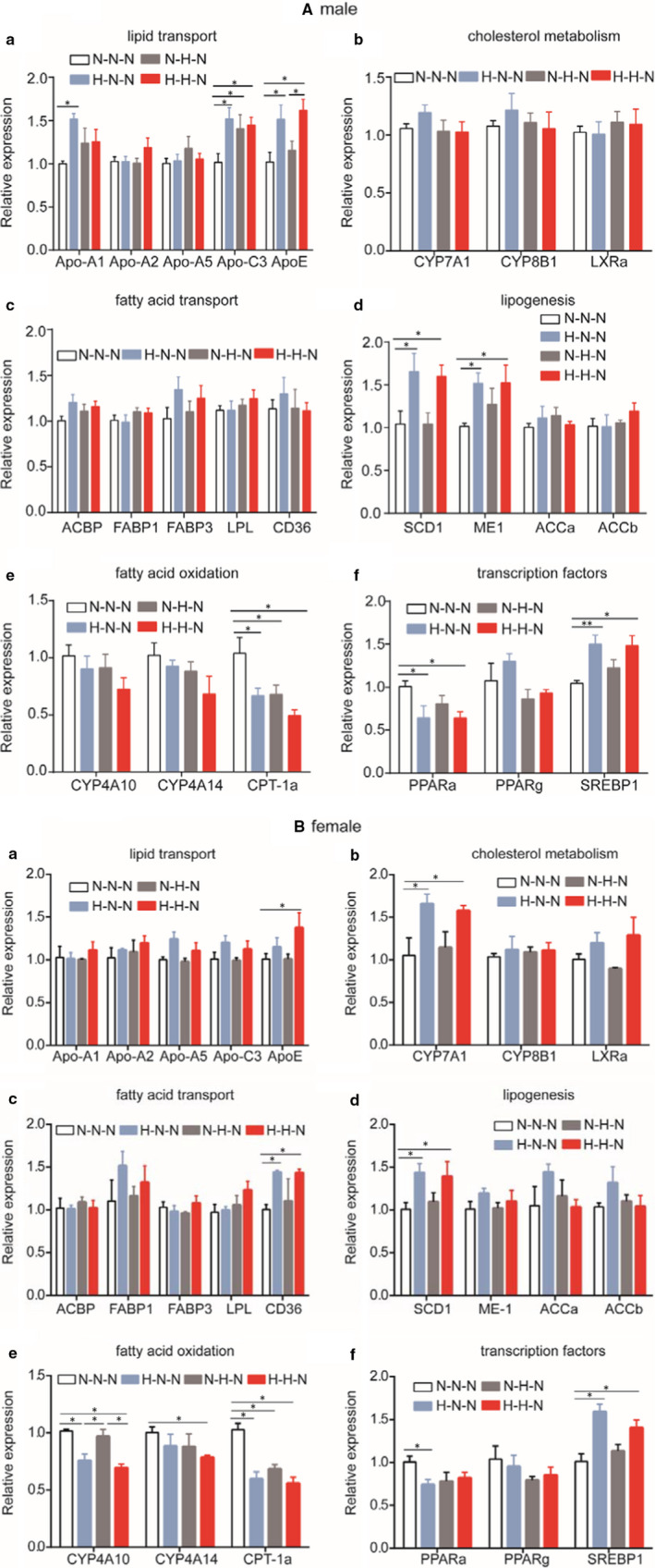
Relative expression of genes involved in lipid metabolism disorders in mice at 16 weeks of age. A, expression of genes in livers of F1 male offspring at 16 weeks of age, B, expression of genes in livers of F1 female offspring at 16 weeks of age; a, lipid transport; b, cholesterol metabolism; c, fatty acid transport, d, lipogenesis; e, fatty acid oxidation and f, transcription factors. (n = 5 mice per group). Data are presented as the mean ± SEM *P < .05, **P < .01, significance was determined by ANOVA

We performed quantitative PCR (qPCR) analysis to determine whether a HFD given in early life could alter the expression of lipid metabolism‐related genes in the livers of F1 offspring at 16 weeks of age. We selected key genes with roles in lipid metabolism, including lipid transport, cholesterol metabolism, fatty acid transport, lipogenesis and fatty acid oxidation, as well as transcription factors (Figure 3). We found that male offspring from mothers given a HFD during gestation had significantly increased levels of *Apo‐A1*, *Apo‐C3*, *Apo‐E*, *Scd1*, *Me1* and *Srebp1* and significantly decreased levels of peroxisome proliferator‐activated receptor‐α (*Pparα*) and *Cpt‐1a* (Figure 3A[a–f], male). Female offspring from mothers given a HFD during gestation also had significantly increased levels of *Cyp7A1*, *Scd1* and *Srebp1*, and significantly decreased levels of *Pparα* and *Cpt‐1a* (Figure 3B[a–f], female). These results suggest that gestational HFD exposure alters lipid metabolism–related genes in the liver, and gestation might be a sensitive period of HFD exposure.

### 3.5 Administration of a HFD impaired fatty acid oxidation earlier than lipogenesis at E18.5 day

**FIGURE 4:**
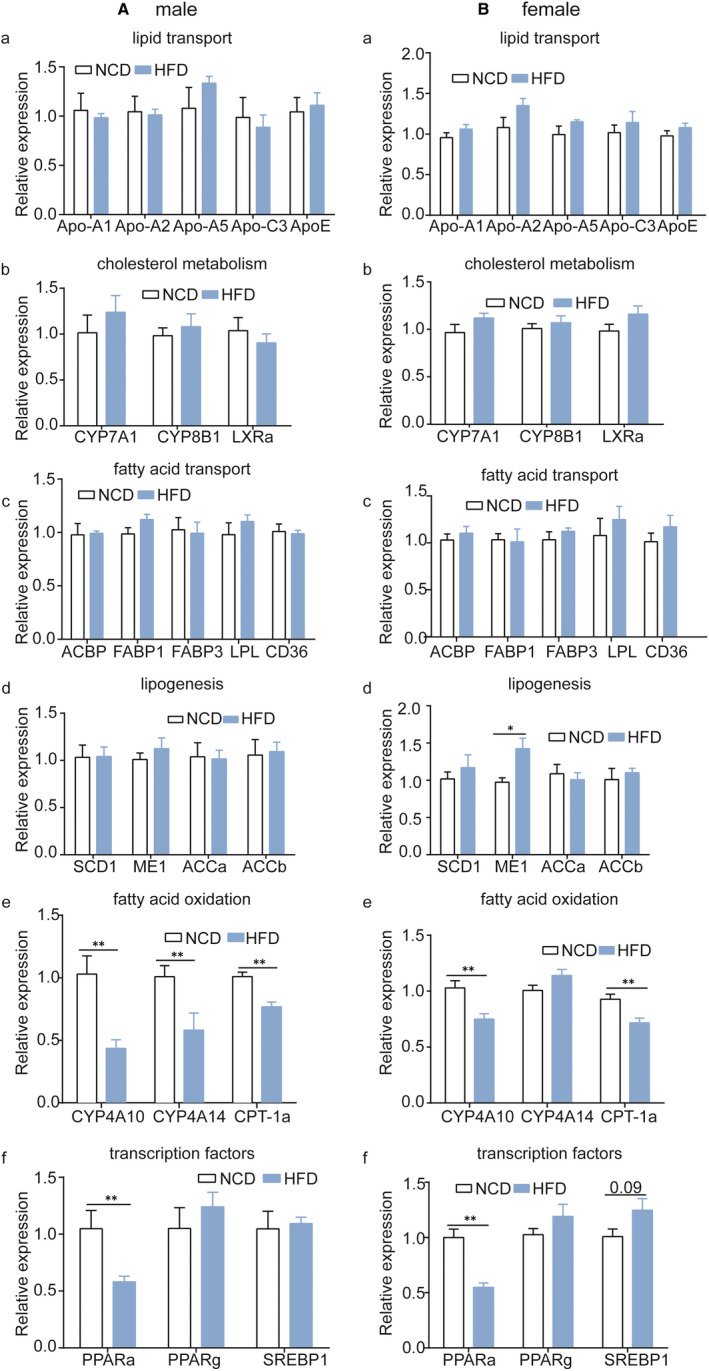
Relative expression of genes involved in lipid metabolism disorders in livers of mice at E18.5 day. A, expression of genes in male foetal livers; B, expression of genes in female foetal livers; a, lipid transport; b, cholesterol metabolism; c, fatty acid transport; d, lipogenesis; e, fatty acid oxidation and f, transcription factors. (male, n = 5 mice per group; female, n = 6 mice per group). Data are presented as the mean ± SEM, unpaired t test: *P *< *.05, **P < .01. NCD, normal chow diet; HFD, high‐fat diet

We performed qPCR analysis to examine the levels of the genes associated with lipid metabolism in the foetal liver at E18.5 day. The genes related to lipid metabolism included those involved in lipid transport, cholesterol metabolism, fatty acid transport and lipogenesis. Compared with the foetuses from mothers given a NCD during gestation, *Cpt‐1a* and *Pparα* mRNA levels were significantly decreased in the livers of both males (Figure 4A[e, f], male) and females (Figure 4B[e, f], female) at E18.5 day from the mothers given a HFD during gestation. These results are consistent with those in the livers of adult offspring (Figure 3) and provide further evidence that gestational HFD exposure alters genes related to lipid metabolism in the liver and gestation is a sensitive period of HFD exposure.

### 3.6 Administration of a HFD reprogrammes DNA methylation patterns by decreasing Tets expression instead of increasing Dnmts expression at E18.5 day

**FIGURE 5:**
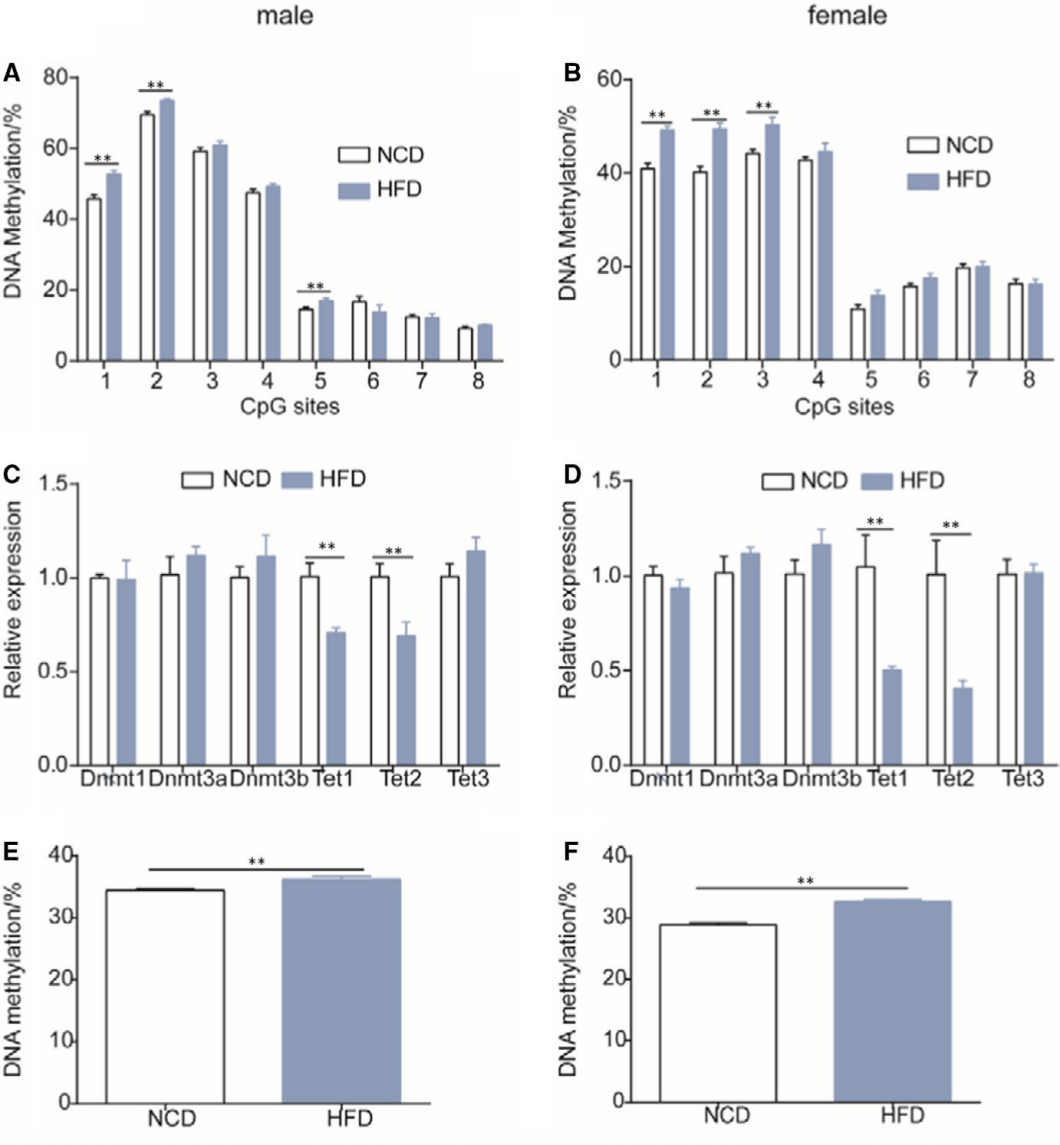
Methylation patterns of *Pparα* and the relative expression of DNA‐modifying enzymes in livers of mice at E18.5 day. A, methylation level of the *Pparα* promoter in livers of male mice at E18.5 day; B, methylation level of the *Pparα* promoter in livers of female mice at E18.5 day; C, relative expression of DNA‐modifying enzymes in livers of male mice at E18.5 day; D, relative expression of DNA‐modifying enzymes in livers of female mice at E18.5 day; E, mean DNA methylation in male mice at E18.5 day; F, mean DNA methylation in male mice at E18.5 day (n = 5 mice per group). Data are presented as the mean ± SEM, unpaired *t* test: *P < .05, **P < .01

Based on our finding of decreased *Pparα* expression in mice at E18.5 day and 16 weeks of age, we further examined the DNA methylation pattern of *Pparα* and the relative mRNA levels of DNA‐modifying enzymes. We found increased methylation in CpG sites of the *Pparα* promoter in the livers of foetuses at E18.5 day from mothers given a HFD during gestation compared to mothers given a NCD during gestation (Figure 5A [male], B [female]). The mean methylation level of the Pparα promoter was significantly higher in offspring of the gestational HFD group than that in offspring of the NCD group (Figure 5E [male], F [female]). These hypermethylation patterns may have been caused by the decreased expression levels of *Tet1* and *Tet2*, because we found that the expression levels *Tet1* and *Tet2* were significantly lower in the liver of foetuses at E18.5 day from mothers given a HFD during gestation than those given a NCD (Figure 5C [male], D [female]), whereas there were no significant differences in the expression levels of the methyltransferases, *Dnmts*, between these two groups (Figure 5C [male], D [female]). These results indicate that a HFD given during gestation induces hypermethylation of *Pparα* by decreasing the expression of *Tet1* and *Tet2*.

### 3.7 Administration of HFD induces hypermethylation of *Pparα* by decreasing Tets expression in offspring at 16 weeks of age

**FIGURE 6:**
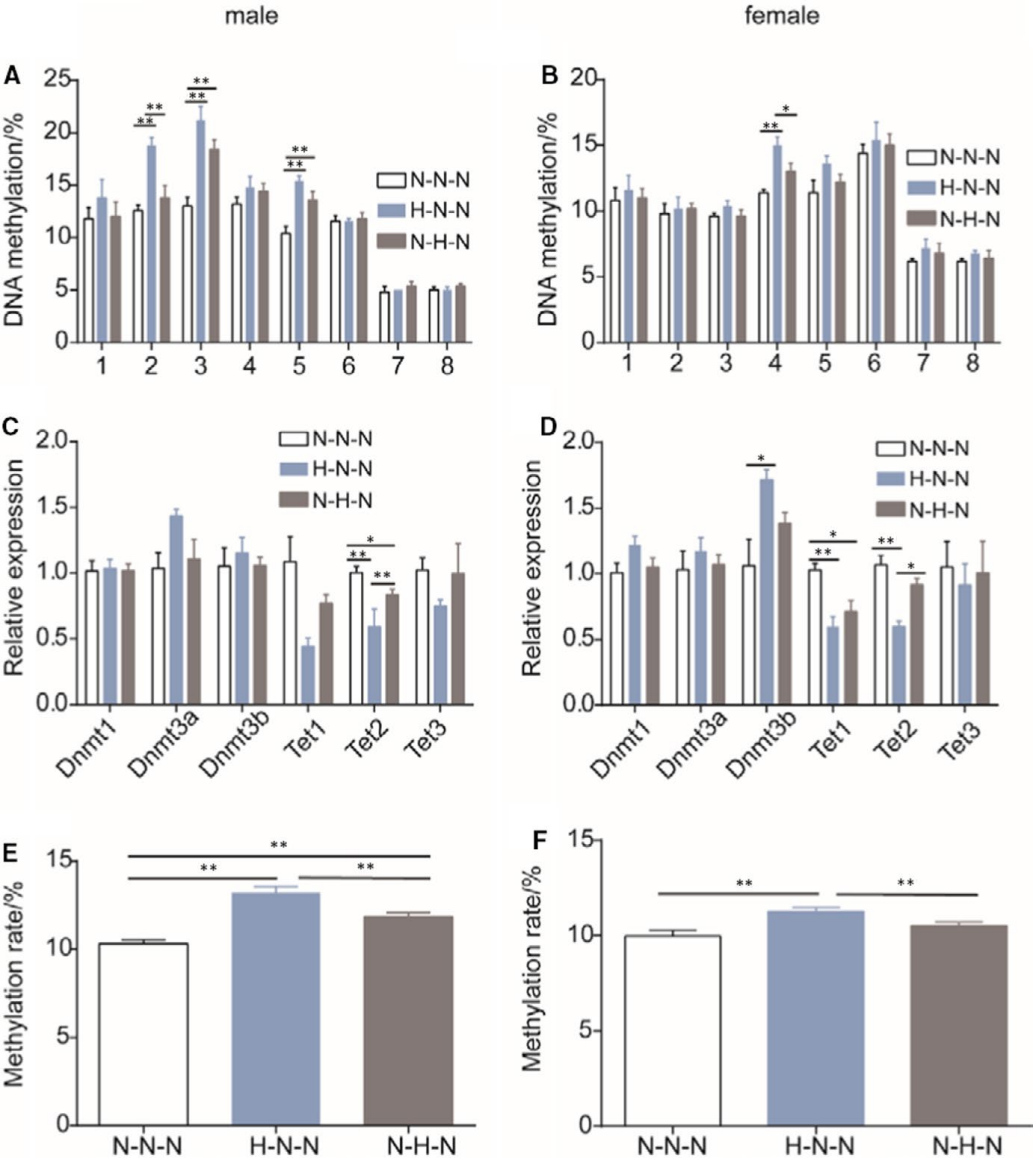
Methylation patterns of *Pparα* and the relative expression of DNA‐modifying enzymes in livers of mice at 16 weeks of age. DNA methylation levels of the *Pparα* promoter in livers of A, male and B, female mice given a normal chow diet after weaning (male, n = 5 mice per group; female, n = 6 mice per group), relative expression of DNA‐modifying enzymes in livers of C, male and D, female mice, E, mean DNA methylation in male mice; F, mean DNA methylation in female mice (male, n = 5 mice per group; female, n = 6 mice per group). Data are presented as the mean ± SEM, significance determined by ANOVA. *P < .05, **P < .01

We examined the DNA methylation patterns of *Pparα* in male and female offspring at 16 weeks of age. Similar to the findings in foetuses, we found increased methylation in CpG sites of the *Pparα* promoter in the livers of offspring at 16 weeks of age from mothers given a HFD during gestation than that from mothers given a NCD (Figure 6A [male], B [female]). The mean methylation level of the Pparα promoter was significantly higher in offspring of the gestational HFD group than those of the NCD group (Figure 6E [male], F [female]). We also detected a lower expression level of *Tet2* in the livers of male offspring (Figure 6C [male]) and lower expression levels of *Tet1* and *Tet2* in the livers of female offspring (Figure 6D [female]) at 16 weeks of age from mothers given a HFD during gestation than those given a NCD, whereas there were no significant differences in the expression levels of *Dnmt1* and *Dnmt3a* between these two groups (Figure 6C [male], D [female]). These results provide further evidence that a HFD given during gestation induces hypermethylation of *Pparα* by decreasing the expression of *Tet1* and *Tet2*.

## 4 DISCUSSION

‘Mismatch'nutritional changes and/or a sedentary lifestyle can increase the risk of obesity.^11^ Previous studies have shown that a maternal HFD‐ or self‐HFD‐induced obesity leads to different metabolism disorders.^12^, ^13^, ^14^ Here, we provide evidence to support two fundamental principles of programming: sensitivity and duration. We found that gestation was the most sensitive period to induce obesity in late life, and there was no difference between the sexes in chance of obesity. Furthermore, we found that lactation and administration of a HFD post‐weaning increased the incidence of lipid metabolism disorders and obesity in offspring.

We speculated that the DNA demethylation level of *Pparα* associated with lipid metabolism disorders and obesity in offspring. The altered DNA methylation pattern of *Pparα* may have been caused by aberrant demethylation, which was attributed to the decreased expression of *Tets* that may have been triggered by a maternal HFD during gestation. Consistently, we found that the aberrant demethylation of *Pparα* was associated with lipid metabolism disorders and obesity in offspring. Mechanistically, the hypermethylation of the *Pparα* promoter is attributed to decreased DNA‐modifying enzymes of *Tets* in offspring from mothers given a HFD during gestation, which may have disrupted demethylation.

Given that different developmental processes take place at different stages, we speculated that the various programming mechanisms were also related to the time point of exposure. However, our results suggest that gestation is the most sensitive period for lipid metabolism disorders and obesity after the administration of a HFD. There are different windows of opportunity for the programming of epigenetically labile genes. Some studies support the alteration of epigenetic status during development as an important cause induced adult obesity.^15^, ^16^ Gestation is considered as the most sensitive period because high DNA synthesis and DNA methylation patterns are established for normal tissue development during the embryonic period.^17^ These two reprogramming events are the times when the epigenetic state changes most widely in the life cycle.

The administration of a HFD during lactation may induce obesity in mice, consistent with the results of previous studies.^18^, ^19^ Sexual dimorphism is a common research area in clinical trials and basic medical research.^20^ In this study, we did not observe sexual dimorphism in lipid metabolism and obesity. One possible reason is that diet‐induced obesity in mice varies by age of onset,^21^ and oestrogen in adult females may have caused a difference between the sexes. Another possibility is that there are significant differences in adipose tissue distribution between males and females,^22^, ^23^ and women typically have greater fat accumulation in gluteal‐femoral depots.^24^

Lipid metabolism mainly involves lipogenesis and fatty acid catabolism. In this study, we revealed that impaired fatty acid oxidation was initiated at pregnancy much earlier than lipogenesis, and the decreased expression of the upstream transcription factor *Pparα* started at gestation. Previous studies have reported that HFD exposure can disrupt lipogenesis and fatty acid oxidation,^25^, ^26^ but they did not identify the initiating factors. In this study, we mainly investigated whether the administration of a HFD during pregnancy would alter the expression of lipid metabolism‐related genes in the livers of offspring. We found that the levels of several lipid metabolism‐related genes were altered in offspring from mothers given a HFD during gestation. Among these genes, decreased *Pparα* expression was not only observed in livers of offspring but also in livers of foetuses from mothers given a HFD during gestation. PPARα is a key regulator of fatty acid oxidation in mice.^27^ Hepatocyte‐specific PPARα deficiency can lead to hepatic lipid accumulation.^28^ PPARα can also stimulate the transcription of fatty acid β‐oxidation relative genes.^29^ In the present study, the administration of a HFD during pregnancy led to decreased expression of *Pparα* in foetal livers, possibly by inhibiting the demethylation of *Pparα*‐dependent fatty acid oxidation–related genes and ultimately inhibiting lipid catabolism and inducing obesity.

There is increasing evidence that environmental stress can affect gene transcription, and gene transcription rates can be regulated through DNA methylation levels. Several studies have hypothesized that DNA methylation is fully established by the time of birth.^30^, ^31^, ^32^ However, many fatty acid β‐oxidation‐related genes undergo DNA demethylation with increased mRNA expression in the postnatal mouse liver.^33^ DNA methylation is a gene transcription silencing mechanism. In this study, we speculated that the relationship between the reduced expression of *Tets* and the increased methylation level of *Pparα* may have started during gestation, thereby leading to lipid metabolism disorders and obesity in offspring later in life. Furthermore, it has been reported that DNA methylation patterns of metabolism‐related genes in the liver change dynamically in early life, thereby activating hepatic metabolic processes to adapt to the nutritional environment.^34^, ^35^, ^36^

The administration of a HFD during gestation down‐regulated the levels of *Tet1* and *Tet2* in offspring at E18.5 days, whereas the *Dnmts* level was not altered. TET2 is necessary for cell development,^37^ and postnatal demethylation in the liver was mediated by TET2.^38^ Reduced expression of *Tet1* and *Tet2* may associate with an enhanced DNA methylation level of *Pparα*, and decreased expression of *Tets* may compromise demethylation. Previous studies have reported that DNA demethylation is PPARα dependent,^39^ and PPARα mediates DNA demethylation of fatty acid β‐oxidation genes.^34^ In the present study, a gestational HFD reduced *Tet1* and *Tet2* expression, thereby resulting in hypermethylation of hepatic *Pparα* and decreased PPARα expression. Finally, reduced PPARα expression increased the risk of obesity in offspring from mothers given a HFD.

In summary, we found that a HFD given during gestation alters lipid metabolism–related genes by altering epigenetic programs. A gestational HFD impairs the demethylation of *Pparα*, thereby inducing obesity in offspring in later life. Early interventions and avoiding exposure should help to prevent lipid metabolic disorders associated with obesity.

## CONFLICT OF INTEREST

The authors declare that they have no conflicts of interest.

## AUTHOR CONTRIBUTIONS

Haiyan Pang, Jianzhong Sheng, Hefeng Huang: designed the experiments. Haiyan Pang, Dandan Ling: performed the animal experiments and collected and analysed data. Yi Cheng, Luyang Jin: performed the animal experiments. Rubab Akbar, Jun Ren, Haiyan Wu, Bin Chen, Yin Zhou, Hong Zhu, Yuzhong Zhou: collected animal materials. Haiyan Pan,Jianzhong Sheng, Hefeng Huang: wrote and edited the manuscript. All authors read and approved the final manuscript. Jianzhong Sheng: is the guarantor of this work and, as much, has full access to all data in the study and takes responsibility for the integrity and accuracy of data analysis.

## Supporting information

Supplementary Material

Table S2

## Data Availability

The data that support the findings of this study are available from the corresponding author upon reasonable request.
