# Supplementary material for: Gestational high‐fat diet impaired demethylation of Ppar α and induced obesity of offspring
Source: J Cell Mol Med. 2021 May 6;25(12):5404–16. doi: 10.1111/jcmm.16551 (PMC8184666; doi:10.1111/jcmm.16551)
Supplement: Supplementary file 1 — Supplementary Material [file JCMM-25-5404-s001.docx]

**Supplemental figure 1**

**Experimental procedures**


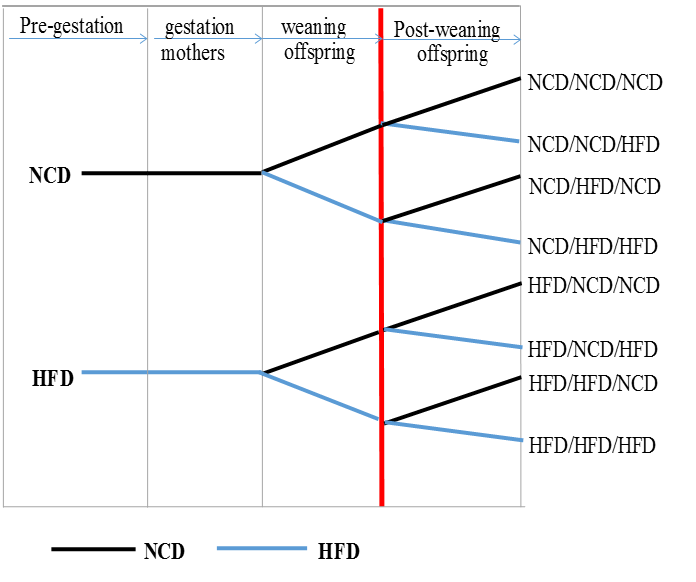


FigureⅠexperimental outline to Experimental outline

**Supplemental figure 2**

We used the Image J software to quantify the fat deposition in the liver. We picked three different pictures in each group represented three different mice.


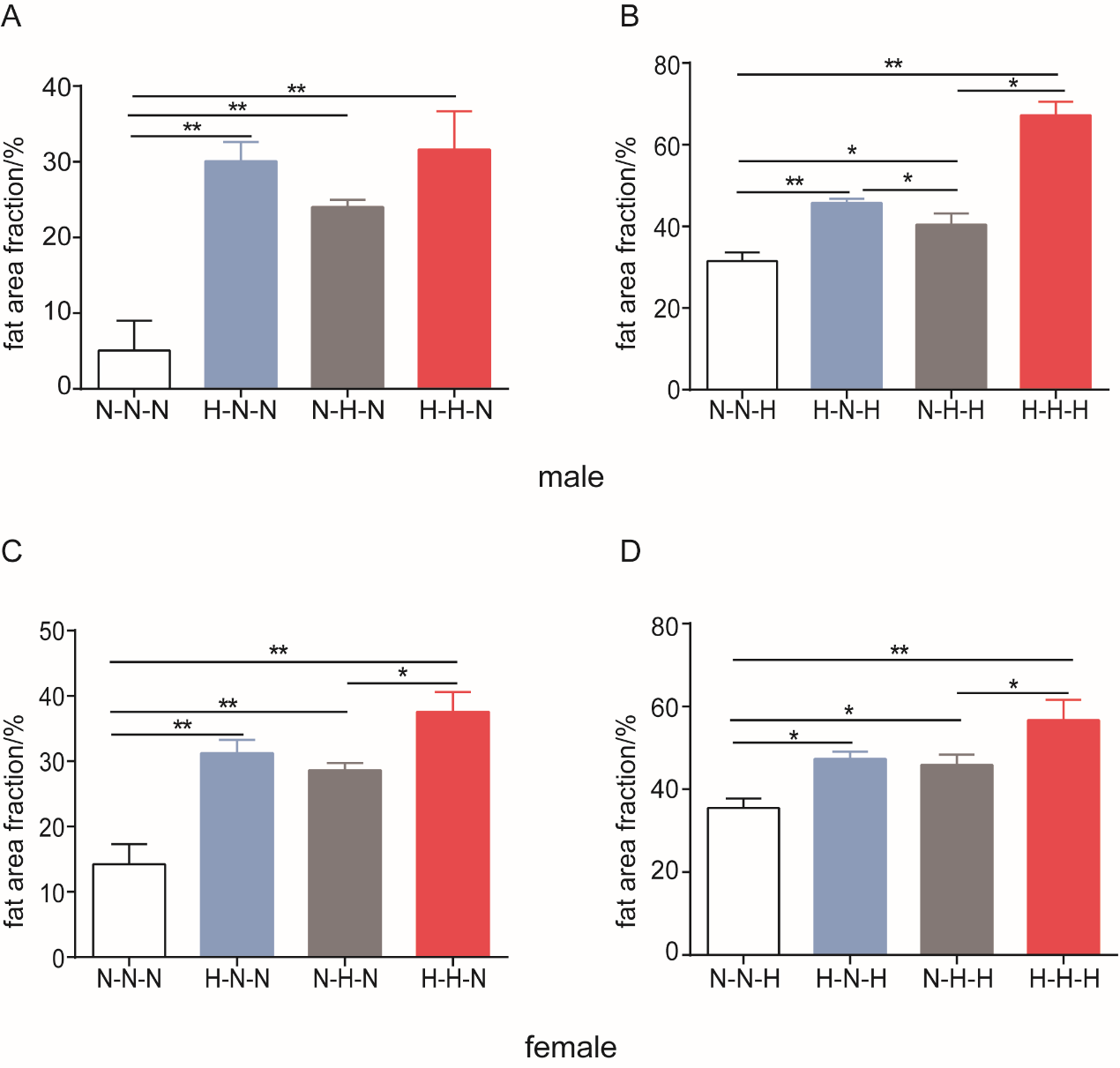


Fat deposition in the liver in F1 offspring at 16 weeks.

(A) fat deposition of F1 male offspring on normal chow diet or (B) high-fat diet after weaning; (C) fat deposition of F1 female offspring on normal chow diet or (D) high-fat diet after weaning. (A): N-N-N=3 vs. H-N-N=3 vs. N-H-N=3 vs. H-H-N=3; (B): N-N-H=3 vs. H-N-H=3 vs. N-H-H=3 vs. H-H-H=3; (C): N-N-N=3 vs. H-N-N=3 vs. N-H-N=3 vs. H-H-N=3; (D): N-N-H=3 vs. H-N-H=3 vs. N-H-H=3 vs. H-H-H=3. Data were presented as mean ± S.E.M. **P*＜0.05, ***P*＜0.01, significance was determined by ANOVA.

Table S1-1 primers are used for RT-PCR. Sequences are presented in the 5’ to 3’ direction

| Gene | Sequence |
| --- | --- |
| *β-actin* | F: GGCTGTATTCCCCTCCATCG  R: CCAGTTGGTAACAATGCCATGT |
| *Gapdh* | F: ACAACTTTGGTATCGTGGAAGG  R: GCCATCACGCCACAGTTTC |
|  |  |
| Lipid biosynthesis |  |
| *Scd1* | F: TGTACGGGATCATACTGGTTCCC  R: CAGCCGAGCCTTGTAAGTTCTGTG |
| *Me1* | F: AACTCTGACTTCGACAGGTATCT  R: CGGAATGCCAAACTGTACTGC |
| *Acca* | F: TTACAGGATGGTTTGGCCTTTC  R: CAAATTCTGCTGGAGAAGCCAC |
| *Accb* | F: CCAGTCTTCCGTGCCTTTGTAC  R: CTCATCCCTCGCTCTGAACG |
|  |  |
| Lipid transport |  |
| *Apo-A1* | F: CTGGCCGTGGCTCTGGTCTTC  R: GCTGTCTTTGACCGCATCCACA |
| *Apo-A2* | F: AATGGTCGCACTGCTGGTAA  R: TTGGCCTTCTCCACCAAATC |
| *Apo-A5* | F: AGGAACTGAGCCATCCACAC  R: GAGAGTCACGCCAGACCAAC |
| *Apo-C3* | F: GGCTGGATGGACAATCACTT  R: TGGTTGGTCCTCAGGGTTAG |
| *Apo-E* | F: GCAGAGCTCCCAAGTCACACAA  R: AGTCGGTTGCGTAGATCCTCCA |
|  |  |
| Cholesterol metabolism |  |
| *Cyp7A1* | F: GCTGAGAGCTTGAAGCACAAGA  R: TTGAGATGCCCAGAGGATCAC |
| *Cyp8B1* | F: TGAATTCTTGAAGGGGATGC  R: CCTTGCTCCCTCAGAAACTG |
| *Lxra* | F: GGAGTGTCGACTTCGCAAATG  R: TCAAGCGGATCTGTTCTTCTGAC |
|  |  |
| Fatty acid transport |  |
| *Acbp* | F: GACCTCAAGGGCAAAGCCAAG  R: TACAGAGGGAGGAGGAGCAG |
| *Fabp1* | F: CCAATTGCAGAGCCAGGAGA  R: CCCCTTGATGTCCTTCCCTTT |
| *Fabp3* | F: AGTCACTGGTGACGCTGGACG  R: AGGCAGCATGGTGCTGAGCTG |
| *Lpl* | F: CCAGAAAAGTGAATCTTGACTTGGT  R: AAGGTCAGAGCCAAGAGAAGCA |
| *Cd36* | F: GTTAAACAAAGAGGTCCTTACACATACAG  R: CAGTGAAGGCTCAAAGATGGC |
|  |  |
| Fatty acid oxidation |  |
| *Cyp4A10* | F: GCCAAATCCAGAGGTGTTTGA  R: AGCAAATTGTTTCCCAATGCA |
| *Cyp4A14* | F: TCAGTCTATTTCTGGTGCTGTTC  R: GAGCTCCTTGTCCTTCAGATGGT |
| *Cpt-1a* | F: AGTGGCCTCACAGACTCCAG  R: GCCCATGTTGTACAGCTTCC |
|  |  |
| Transcription factors |  |
| *Pparα* | F: CCCTGTTTGTGGCTGCTATAATTT  R: GGGAAGAGGAAGGTGTCATCTG |
| *Pparg* | F: CACAATGCCATCAGGTTTGG  R: GCTGGTCGATATCACTGGAGATC |
| *Srebp-1c* | F: CAGCTCAGAGCCGTGGTGA  R: TTGATAGAAGACCGGTAGCGC |

Table S1-2 primers are used for RT-PCR. Sequences are presented in the 5’ to 3’ direction

| Gene | Sequence |
| --- | --- |
| *β-actin* | F: GGCTGTATTCCCCTCCATCG  R: CCAGTTGGTAACAATGCCATGT |
| *Dnmt1* | F: ACCTGGAGAGCAGAAATGGC  R: TCCTCGTAGCCACGGAACTA |
| *Dnmt3a* | F: CAGAGCCGCCTGAAGCC  R: TCTTCCTTGCCACGGTTCTC |
| *Dnmt3b* | F: TCAGAAGGCTGGAGACCTCCCTCTT  R: TTCAGTGACCAGTCCTCAGACACGAA |
| *Tet1* | F: AATGGGCCAACCAGGAAGAG  R: GTTGTGTGAACCTGATTTATTGTGG |
| *Tet2* | F: ATATTGATGCGGAGGCGAGG  R: CAAATCCTACAGGGCAGCCA |
| *Tet3* | F: TCCGGGAACTCATGGAGGAT  R: GAACTCTTCCCCTCCTTGCC |
