# Supplementary material for: Gestational high‐fat diet impaired demethylation of Ppar α and induced obesity of offspring
Source: J Cell Mol Med. 2021 May 6;25(12):5404–16. doi: 10.1111/jcmm.16551 (PMC8184666; doi:10.1111/jcmm.16551)
Supplement: Supplementary file 2 — Table S2 [file JCMM-25-5404-s002.docx]

Table S2 Pyrosequencing primers of *Pparα*. (primers used at 100nM)

| Primers | [Btn]indicates biotinylated primer | temperature | Sequence to analyze (5’-3’) |
| --- | --- | --- | --- |
| CpG 1 to 4 | F: TTGGTTAGGAAATGTGGGGGAAG  R: [Btn] ACACATACAACAAAAACACCATCTATACA  S: GGATGAGTGTGGTTAA | 55℃ | TTTGTAGTTYGTGGGTTATTTATATTTTAGGATAGATTAATATATTTGTAGGTGATATTTAGYGTGAATTAAAGTATATGGTTTAAGATTTTTTAGTGGTTATTAATGTGTTAAYGAGGGGATATAAGAATATTAAYGGTGTTTATTTAGTTTTGTATAGAT |
| CpG 5 to 8 | F:  GGGTTTGGTTTTTAGTTTTTAGGGTAAGGA  R: [Btn] AAATATATACTATACCAACCCTCCTATC  S: AGGGTAAGGAGATGT | 56℃ | GYGTTGAAAGTTTTTTTTTTTTAGTAGGTTTTTTTGGGYGTTTATYGTTATAAATAGTATAGTGGTAGGTTTAGYGTGAGGATAGATAGGAGGGTTGGTA |
